# Supplementary material for: A Comprehensive Review of Small-Molecule Inhibitors Targeting Bruton Tyrosine Kinase: Synthetic Approaches and Clinical Applications
Source: Molecules. 2023 Dec 11;28(24):8037. doi: 10.3390/molecules28248037 (PMC10746017; doi:10.3390/molecules28248037)
Supplement: Supplementary file 1 [file molecules-28-08037-s001.zip › molecules-2723644-supplementary.pdf]

Supporting Information

A Comprehensive Review of Small-Molecule Inhibitors Targeting Bruton Tyrosine Kinase: Synthetic Approaches and Clinical Applications

Qi Zhang [1], Changming Wen [1], Lijie Zhao [4,*] and Yatao Wang [2,3,*]

[1] Nanyang Central Hospital, Nanyang 473000, China; zhangqizzu@126.com (Q.Z.); 13838729696@sina.com (C.W.)
[2] First People's Hospital of Shangqiu, Shangqiu 476100, China
[3] Department of Orthopedics, China-Japan Union Hospital, Jilin University, Changchun 130033, China
[4] The Rogel Cancer Center, Department of Internal Medicine, University of Michigan, Ann Arbor, MI 48109, USA
* Corresponding: lijiez@sioc.ac.cn (L.Z.); yataowangjlu@outlook.com (Y.W.)

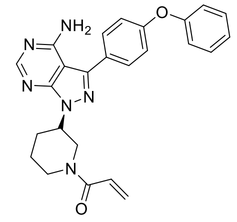

Ibrutinib

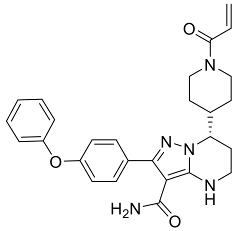

Zanubrutinib

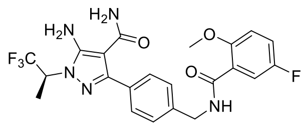

Pirtobrutinib

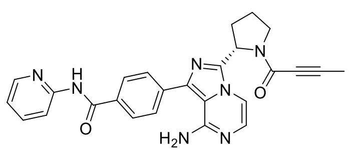

Acalabrutinib

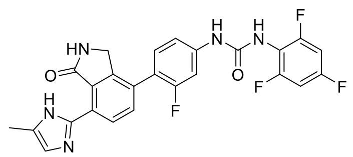

Luxeptinib

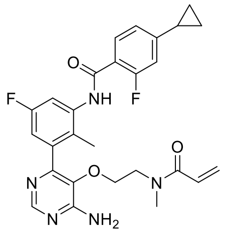

Remibrutinib

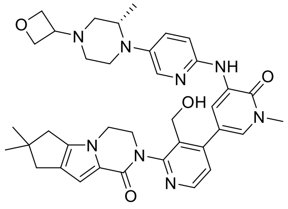

Fenebrutinib

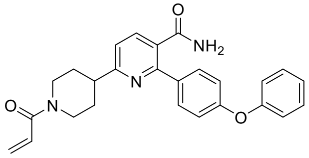

Orelabrutinib

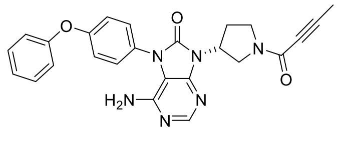

Tirabrutinib

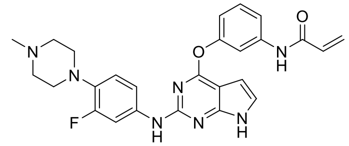

Abivertinib

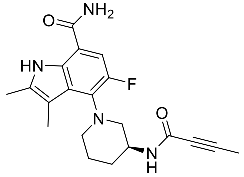

Branebrutinib

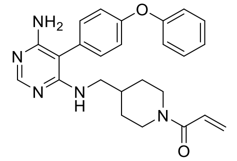

Evobrutinib

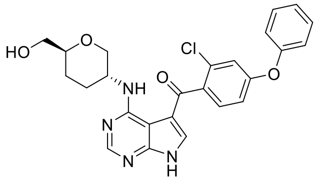

Nemtabrutinib

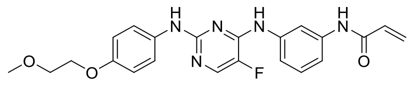

Spebrutinib

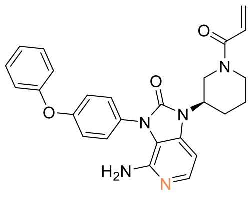

Tolebrutinib

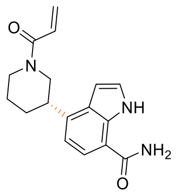

Elsubrutinib

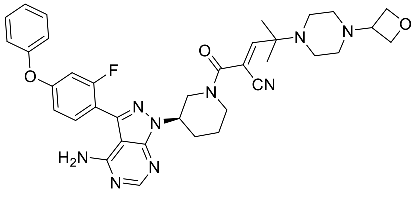

Rilzabrutinib

Figure S1. Chemical structures of representative BTK inhibitors in the clinic.

Table S1. Representative BTK inhibitors in various clinical stages.

NO	Drug	Company	Indications	IC_{50}	mechanism of action	Phase	Clinical trial ID	Status
1	Ibrutinib	Pharmacyclics Llc	Mantle cell lymphoma (MCL), Waldenström's macroglobulinemia (WM), chronic lymphocytic leukemia (CLL), marginal zone B-cell lymphoma (MZBL) and graft versus host disease (GvHD)	0.5 nM	irreversible			Approved (2013)
2	Acalabrutinib	AstraZeneca and Acerta Pharma Llc	MCL and CLL	3-5.1 nM	irreversible			Approved (2017)
3	Zanubrutinib	BeiGene	MCL, MZBL, CLL and WM	0.3 nM	irreversible			Approved (2019)
4	Tirabrutinib Hydrochlorid e	Ono Pharmaceutical.	lymphoma	6.8 nM	irreversible			Approved (2020)
5	Orelabrutinib	Beijing InnoCare Pharma Tech	MCL, MZBL and CLL	1.6 nM	irreversible			Approved (2020)
6	Pirtobrutinib	Redx Pharma	MCL	3.15 nM	reversible			Approved (2023)
7	Luxeptinib	Aptose Biosciences Inc.	A Study of CG-806 in Patients with Relapsed or Refractory CLL/SLL or Non-Hodgkin's Lymphomas	32 nM	reversible	Phase 1	NCT03893682	Active, not recruiting
		Aptose Biosciences Inc.	A Study of CG-806 in Patients with Relapsed or Refractory AML or Higher-Risk MDS			Phase 1	NCT04477291	Recruiting
8	Spebrutinib	Celgene	Efficacy and Safety Study of CC-292 Versus Placebo as Co-therapy With Methotrexate in Active Rheumatoid Arthritis	0.5 nM	irreversible	Phase 2	NCT01975610	Completed

		Celgene	Novel Combinations of CC-122, CC-223, CC-292, and Rituximab in Diffuse Large B-cell Lymphoma and Follicular Lymphoma			Phase 1	NCT02031419	Active, not recruiting
9	Branebrutinib	Bristol-Myers Squibb	Study to Assess Safety and Effectiveness of Branebrutinib Treatment in Participants with Active Systemic Lupus Erythematosus or Primary Sjögren's Syndrome, or Branebrutinib Treatment Followed by Open-label Abatacept Treatment in Study Participants with Active Rheumatoid Arthritis	0.1 nM	irreversible	Phase 2	NCT04186871	Completed
		Bristol-Myers Squibb	Study to Assess the Effect of Branebrutinib on the Drug Levels of Rosuvastatin in Healthy Participants			Phase 1	NCT04515628	Completed
		Bristol-Myers Squibb	A Study to Assess Relative Bioavailability of Branebrutinib, From a Tablet Formulation to the Capsule Formulation, the Effect of Food on the Bioavailability of Branebrutinib from a Tablet Formulation, and the Safety and Drug Levels of Branebrutinib from a Tablet Formulation in Healthy Participants			Phase 1	NCT05303220	Completed
		Bristol-Myers Squibb	A Study of BMS-986166 or Branebrutinib for the Treatment of Participants with Atopic Dermatitis			Phase 2	NCT05014438	Completed
		Bristol-Myers Squibb	A Study of BMS-986195 in Healthy Male Subjects			Phase 1	NCT03245515	Completed
		Bristol-Myers Squibb	Safety, Tolerability and Relative Bioavailability Study of BMS-986195 in Healthy Subjects			Phase 1	NCT02705989	Completed
		Bristol-Myers Squibb	Effects of Concomitant Administration of BMS-986195 on Methotrexate, Caffeine, Montelukast,			Phase 1	NCT03131973	Completed

			Flurbiprofen, Omeprazole, Midazolam, Digoxin, and Pravastatin					
		Bristol-Myers Squibb	The Effect of BMS-986195 Combined with an Oral Contraceptive (Ethinyl Estradiol/Norethindrone) in Healthy Female Patients			Phase 1	NCT03262740	Completed
10	Elsubrutinib	AbbVie	A Study to Investigate the Safety and Efficacy of Elsubrutinib and Upadacitinib Given Alone or in Combination in Participants with Moderately to Severely Active Systemic Lupus Erythematosus (SLE)	0.18 μM	irreversible	Phase 2	NCT03978520	Completed
		AbbVie	A Study of the Safety of Oral Elsubrutinib Capsules and Oral Upadacitinib Tablets Given Alone or in Combination (ABBV-599) for Adult Participants with Moderately to Severely Active Systemic Lupus Erythematosus to Assess Change in Disease State			Phase 2	NCT04451772	Active, not recruiting
		AbbVie	A Study in Rheumatoid Arthritis Patients Who Have Completed a Preceding Study With ABBV-105 Given Alone or in Combination with Upadacitinib			Phase 2	NCT03823378	Terminated
		AbbVie	A Study to Investigate the Safety and Efficacy of ABBV-105 Alone or in Combination with Upadacitinib (ABBV-599 Combination) in Participants with Active Rheumatoid Arthritis			Phase 2	NCT03682705	Completed
11	Evobrutinib	Merck KGaA, Darmstadt, Germany	Effect of Renal Impairment on Evobrutinib Pharmacokinetics (PK)	9 nM	irreversible	Phase 1	NCT03436394	Completed
		Merck Healthcare	Effect of Meal Composition and Timing on			Phase	NCT03934502	Completed

KGaA, Darmstadt, Germany, an affiliate of Merck KGaA, Darmstadt, Germany	Evobrutinib Bioavailability	1		
Merck Healthcare KGaA, Darmstadt, Germany, an affiliate of Merck KGaA, Darmstadt, Germany	Relative Bioavailability (rBA) of Evobrutinib Intended Commercial and Clinical Tablets, and Effect of Food on Intended Commercial Tablets	Phase 1	NCT04314024	Completed
Merck KGaA, Darmstadt, Germany	Human Absorption, Distribution, Metabolism and Excretion (ADME) of [14C]-Evobrutinib	Phase 1	NCT03725072	Completed
EMD Serono Research & Development Institute, Inc.	A Study of Efficacy and Safety of M2951 in Participants with Relapsing Multiple Sclerosis	Phase 2	NCT02975349	Active, not recruiting
Merck Healthcare KGaA, Darmstadt, Germany, an affiliate of Merck KGaA, Darmstadt, Germany	DDI Study of Evobrutinib and Carbamazepine	Phase 1	NCT05248945	Completed
Merck Healthcare KGaA, Darmstadt,	Study Comparing Pharmacokinetics of Different Formulations of Evobrutinib in Healthy Participants	Phase 1	NCT05245396	Completed

Germany, an affiliate of Merck KGaA, Darmstadt, Germany				
EMD Serono Research & Development Institute, Inc.	Phase IIb Study of Evobrutinib in Subjects with Rheumatoid Arthritis	Phase 2	NCT03233230	Completed
EMD Serono Research & Development Institute, Inc.	Study of Evobrutinib in Participants With RMS	Phase 3	NCT04032171	Terminated
EMD Serono Research & Development Institute, Inc.	A Phase II Study of M2951 in SLE	Phase 2	NCT02975336	Terminated
Merck Healthcare KGaA, Darmstadt, Germany, an affiliate of Merck KGaA, Darmstadt, Germany	Drug-Drug Interaction Study of Evobrutinib and Transporter Substrates	Phase 1	NCT05064488	Completed
Merck Healthcare KGaA, Darmstadt, Germany, an affiliate of Merck	Study of Evobrutinib in Participants with RMS (evolutionRMS 2)	Phase 3	NCT04338061	Active, not recruiting

KGaA, Darmstadt, Germany				
Merck Healthcare KGaA, Darmstadt, Germany, an affiliate of Merck KGaA, Darmstadt, Germany	Study of Evobrutinib in Participants with RMS (evolutionRMS 1)	Phase 3	NCT04338022	Active, not recruiting
EMD Serono Research & Development Institute, Inc.	Safety and Efficacy Study of M2951 in Participants with Rheumatoid Arthritis	Phase 2	NCT02784106	Completed
EMD Serono Research & Development Institute, Inc.	Study of Evobrutinib in Participants with Relapsing Multiple Sclerosis (RMS)	Phase 3	NCT04032158	Terminated
Merck Healthcare KGaA, Darmstadt, Germany, an affiliate of Merck KGaA, Darmstadt, Germany	Effect of Hepatic Impairment on M2951 (BTK Inhibitor) PK	Phase 1	NCT04546789	Completed
EMD Serono Research & Development Institute, Inc.	MSC2364447C Phase 1b in Systemic Lupus Erythematosus	Phase 1	NCT02537028	Completed

		Merck Healthcare KGaA, Darmstadt, Germany, an affiliate of Merck KGaA, Darmstadt, Germany	Drug-drug Interaction Study of Evobrutinib with Midazolam in Healthy Participants			Phase 1	NCT04697511	Completed
12	Fenebrutinib	Hoffmann-La Roche	Study Investigating a Single Oral Dose of Fenebrutinib in Healthy Volunteers	Ki = 0.91 nM	reversible	Phase 1	NCT03596632	Completed
		Hoffmann-La Roche	A Study to Investigate the Efficacy of Fenebrutinib in Relapsing Multiple Sclerosis (RMS)			Phase 2	NCT05119569	Active, not recruiting
		Genentech, Inc.	A Study to Evaluate the Long-term Safety and Efficacy of Fenebrutinib in Participants Previously Enrolled in a Fenebrutinib Chronic Spontaneous Urticaria (CSU) Study			Phase 2	NCT03693625	Terminated
		Hoffmann-La Roche	Study to Evaluate the Efficacy and Safety of Fenebrutinib Compared with Teriflunomide in Relapsing Multiple Sclerosis (RMS)			Phase 3	NCT04586023	Recruiting
		Hoffmann-La Roche	A Study to Evaluate the Efficacy and Safety of Fenebrutinib Compared with Teriflunomide in Relapsing Multiple Sclerosis (RMS)			Phase 3	NCT04586010	Recruiting
		Hoffmann-La Roche	A Study to Evaluate the Efficacy and Safety of Fenebrutinib Compared with Ocrelizumab in Adult Participants with Primary Progressive Multiple Sclerosis			Phase 3	NCT04544449	Active, not recruiting
		Genentech, Inc.	A Study of GDC-0853 in Patients with Resistant B-Cell Lymphoma or Chronic Lymphocytic Leukemia			Phase 1	NCT01991184	Completed

		Genentech, Inc.	A Study of GDC-0853 in Participants with Refractory Chronic Spontaneous Urticaria (CSU)			Phase 2	NCT03137069	Completed
		Genentech, Inc.	A Study of the Safety and Efficacy of GDC-0853 in Participants with Moderate to Severe Active Systemic Lupus Erythematosus			Phase 2	NCT02908100	Completed
13	Nemtabrutinib	Merck Sharp & Dohme LLC	A Clinical Study of Nemtabrutinib in Japanese Participants with Hematological Malignancies (MK-1026-002)	0.85 nM	reversible	Phase 1	NCT05673460	Recruiting
		Merck Sharp & Dohme LLC	A Study of Nemtabrutinib (MK-1026) in China Participants with Relapsed or Refractory Hematologic Malignancies (MK-1026-005)			Phase 1	NCT05347225	Active, not recruiting
		Merck Sharp & Dohme LLC	Efficacy and Safety of Nemtabrutinib (MK-1026) in Participants with Hematologic Malignancies (MK-1026-003)			Phase 2	NCT04728893	Recruiting
		ArQule, Inc. (a wholly owned subsidiary of Merck Sharp and Dohme, a subsidiary of Merck & Co., Inc.)	A Study of Nemtabrutinib (MK-1026) in Participants with Relapsed or Refractory Hematologic Malignancies (ARQ 531-101/MK-1026-001)			Phase 1 Phase 2	NCT03162536	Active, not recruiting
		Merck Sharp & Dohme LLC	A Study of Nemtabrutinib Plus Venetoclax vs Venetoclax + Rituximab (VR) in Second-line (2L) + Relapsed/Refractory (R/R) Chronic Lymphocytic Leukemia/Small Lymphocytic Lymphoma (CLL/SLL) (MK-1026-010/BELLWAVE-010)			Phase 3	NCT05947851	Recruiting

			NEW					
		Merck Sharp & Dohme LLC	A Study of Nemtabrutinib vs Chemoimmunotherapy for Participants with Previously Untreated Chronic Lymphocytic Leukemia/Small Lymphocytic Lymphoma (CLL/SLL) Without TP53 Aberrations (MK-1026-008, BELLWAVE-008)			Phase 3	NCT05624554	Recruiting
		Merck Sharp & Dohme LLC	A Study of Zilovertamab Vedotin (MK-2140) as Monotherapy and in Combination in Participants with Aggressive and Indolent B-cell Malignancies (MK-2140-006)			Phase 2	NCT05458297	Recruiting
14	Remibrutinib	Novartis Pharmaceuticals	Study of Efficacy, Safety and Tolerability of Remibrutinib in Adult Participants with an Allergy to Peanuts	0.23 μM	irreversible	Phase 2	NCT05432388	Recruiting
		Novartis Pharmaceuticals	A Phase 3 Study of Efficacy and Safety of Remibrutinib in the Treatment of CSU in Adults Inadequately Controlled by H1- Antihistamines			Phase 3	NCT05032157	Active, not recruiting
		Novartis Pharmaceuticals	An Extension Study of Long-term Efficacy, Safety and Tolerability of Remibrutinib in Chronic Spontaneous Urticaria Patients Who Completed Preceding Studies with Remibrutinib			Phase 3	NCT05513001	Recruiting
		Novartis Pharmaceuticals	A Phase 3 Study of Efficacy and Safety of Remibrutinib in the Treatment of CSU in Adults Inadequately Controlled by H1 Antihistamines			Phase 3	NCT05030311	Recruiting
		Novartis Pharmaceuticals	Efficacy and Safety of Remibrutinib Compared to Teriflunomide in Participants with Relapsing Multiple Sclerosis (RMS)			Phase 3	NCT05156281	Recruiting

Novartis Pharmaceuticals	A Study to Investigate Efficacy, Safety, and Tolerability of Remibrutinib Compared with Placebo in Adults with CINDU Inadequately Controlled by H1-antihistamines	Phase 3	NCT05976243	Not yet recruiting
Novartis Pharmaceuticals	Efficacy and Safety of Remibrutinib Compared to Teriflunomide in Participants with Relapsing Multiple Sclerosis	Phase 3	NCT05147220	Recruiting
Novartis Pharmaceuticals	A Phase 2 Study to Evaluate the Safety and Efficacy of LOU064 in Patients with Moderate to Severe Sjögren's Syndrome	Phase 2	NCT04035668	Terminated
Novartis Pharmaceuticals	A Safety and Efficacy Study of Remibrutinib in the Treatment of CSU in Japanese Adults Inadequately Controlled by H1-antihistamines	Phase 3	NCT05048342	Active, not recruiting
Novartis Pharmaceuticals	A Multicenter, Open-label Phase 3 Study: Ambulatory Blood Pressure Monitoring in Adult Patients with Chronic Spontaneous Urticaria Inadequately Controlled by H1-antihistamines Treated with Remibrutinib up to 12 Weeks.	Phase 3	NCT05795153	Recruiting
Novartis Pharmaceuticals	A Study to Investigate the Pharmacokinetics and Safety of Remibrutinib in Participants with Hepatic Impairment Compared with Matched Healthy Participants	Phase 1	NCT05753592	Recruiting
Novartis Pharmaceuticals	A Phase 3b Study to Assess the Efficacy, Safety, and Tolerability of Remibrutinib in Comparison to Placebo and With Omalizumab as Active Control in CSU Adult Patients	Phase 3	NCT06042478	Not yet recruiting

		Novartis Pharmaceuticals	24 Weeks Double-blind Randomized Placebo-controlled Trial to Evaluate Efficacy, PK, Safety of LOU064 in Adolescents (12 - <18) With CSU and Inadequate Response to H1-antihistamine Followed by Optional 3 Years Open-label Extension and an Optional 3 Years Safety Long-term Treatment-free Follow-up			Phase 3	NCT05677451	Recruiting
15	Rilzabrutinib	Principia Biopharma, a Sanofi Company	Food Effect and Relative Bioavailability Study of Rilzabrutinib in Healthy Participants	1.3 nM	reversible	Phase 1	NCT04748926	Completed
		Sanofi	Efficacy, Safety and Pharmacokinetics of Rilzabrutinib in Patients with Warm Autoimmune Hemolytic Anemia (wAIHA)			Phase 2	NCT05002777	Recruiting
		Principia Biopharma, a Sanofi Company	Open Label Two-Arm Study to Evaluate Rilzabrutinib in IgG4-Related Disease Patients			Phase 2	NCT04520451	Active, not recruiting
		Principia Biopharma, a Sanofi Company	Study to Evaluate Rilzabrutinib in Adults and Adolescents with Persistent or Chronic Immune Thrombocytopenia (ITP)			Phase 3	NCT04562766	Recruiting
		Sanofi	Proof of Concept Study of Rilzabrutinib in Adult Patients with Moderate-to-severe Atopic Dermatitis			Phase 2	NCT05018806	Active, not recruiting
		Sanofi	Proof of Concept Study of Rilzabrutinib in Adult Participants with Moderate-to-severe Asthma			Phase 2	NCT05104892	Recruiting
		Sanofi	Rilzabrutinib for the Treatment of Chronic Spontaneous Urticaria in Patients Who Remain Symptomatic Despite the Use of H1 Antihistamine			Phase 2	NCT05107115	Active, not recruiting

		Principia Biopharma, a Sanofi Company	A Study of PRN1008 in Patients with Pemphigus			Phase 3	NCT03762265	Terminated
		Principia Biopharma, a Sanofi Company	A Study of PRN1008 in Adult Patients with Pemphigus Vulgaris			Phase 2	NCT02704429	Completed
		Principia Biopharma, a Sanofi Company	A Study of Rilzabrutinib in Adult Patients with Immune Thrombocytopenia (ITP)			Phase 1 Phase 2	NCT03395210	Active, not recruiting
		Chen Miao	Zanubrutinib in the Treatment of Relapsed/Refractory wAIHA			Phase 2	NCT05922839	Not yet recruiting
16	Tolebrutinib	National Institute of Neurological Disorders and Stroke (NINDS)	Tolebrutinib, a Brain-penetrant Bruton s Tyrosine Kinase Inhibitor, for the Modulation of Chronically Inflamed White Matter Lesions in Multiple Sclerosis	IC50s of 0.4 and 0.7 nM in Ramos B cells and in HMC microglia cells	irreversible	Phase 2	NCT04742400	Active, not recruiting
		Sanofi	Study to Assess the Plasma Concentration of Tolebrutinib Given as a Tablet to Adult Participants with Renal Impairment Compared to Healthy Participants			Phase 1	NCT05282030	Completed
		Sanofi	Study to Assess the Plasma Concentration of			Phase	NCT05283915	Completed

			Tolebrutinib Given as a Tablet to Adult Participants with Mild Hepatic Impairment Compared to Participants with Normal Hepatic Function			1		
		Sanofi	Long Term Safety and Efficacy Study of Tolebrutinib (SAR442168) in Participants with Relapsing Multiple Sclerosis			Phase 2	NCT03996291	Active, not recruiting
		Sanofi	Nonrelapsing Secondary Progressive Multiple Sclerosis (NRSPMS) Study of Bruton's Tyrosine Kinase (BTK) Inhibitor Tolebrutinib (SAR442168)			Phase 3	NCT04411641	Active, not recruiting
		Sanofi	Primary Progressive Multiple Sclerosis (PPMS) Study of Bruton's Tyrosine Kinase (BTK) Inhibitor Tolebrutinib (SAR442168)			Phase 3	NCT04458051	Recruiting
		Sanofi	Study of Drug-drug Interaction of the Effects of Gemfibrozil and Rifampicin on SAR442168 in Healthy Adult Subjects			Phase 1	NCT06064539	Completed
		Sanofi	Efficacy and Safety of Tolebrutinib (SAR442168) Tablets in Adult Participants with Generalized Myasthenia Gravis			Phase 3	NCT05132569	Terminated
		Sanofi	Relapsing Forms of Multiple Sclerosis (RMS) Study of Bruton's Tyrosine Kinase (BTK) Inhibitor Tolebrutinib (SAR442168)			Phase 3	NCT04410991	Active, not recruiting
		Sanofi	Relapsing Forms of Multiple Sclerosis (RMS) Study of Bruton's Tyrosine Kinase (BTK) Inhibitor Tolebrutinib (SAR442168)			Phase 3	NCT04410978	Active, not recruiting
17	Abivertinib	Sorrento Therapeutics, Inc.	Study to Assess Abivertinib in Combination with Abiraterone in Metastatic Castration Resistant	7.68 nM	irreversible	Phase 2	NCT05361915	Suspended

	Prostate Cancer			
Sorrento Therapeutics, Inc.	Study of the Safety and Efficacy of STI-5656 (Abivertinib Maleate) in Subjects Hospitalized Due to COVID-19	Phase 2	NCT04528667	Completed
Sorrento Therapeutics, Inc.	Study of the Efficacy and Safety of STI-5656 (Abivertinib Maleate) in Subjects Hospitalized With COVID-19	Phase 2	NCT04440007	Completed
Hangzhou ACEA Pharmaceutical Research Co., Ltd.	Abivertinib Maleate Versus Geifitinib in Patients with Advanced Non-small Cell Lung Cancer with Sensitive EGFR Mutation	Phase 3	NCT03856697	Not yet recruiting
Sun Yat-sen University	Safety, Tolerability, Pharmacokinetics and Anti-tumour Activity of AC0010 in Advanced Non-Small Cell Lung Cancer	Phase 1 Phase 2	NCT02274337	Unknown status
Guangdong Association of Clinical Trials	Phase II Umbrella Study Directed by Next Generation Sequencing	Phase 2	NCT03574402	Recruiting
Hangzhou ACEA Pharmaceutical Research Co., Ltd.	Clinical Trial of the Efficacy and Safety of AC0010 in the Treatment of EGFR T790M Patients with Advanded NSCLC	Phase 2	NCT03300115	Unknown status
Hangzhou ACEA Pharmaceutical Research Co., Ltd.	A Phase I Study of AC0010 in Patients With CLL/ SLL, MCL, DLBCL and Other NHL	Phase 1	NCT03060850	Unknown status
Hangzhou ACEA Pharmaceutical Research Co., Ltd.	Preliminary Evaluation of Safety and Efficacy by [14C] AC0010 Trail and Subsequent AC0010 Treatment	Phase 1	NCT03053219	Completed

Hangzhou ACEA Pharmaceutical Research Co., Ltd.	Study to Investigate the Absorption, Metabolism and Excretion of [14C] AC0010 in Patients with Advanced NSCLC	Phase 1	NCT03001609	Completed
ACEA Therapeutics, Inc.	Safety, Pharmacokinetic and Preliminary Efficacy Study of AC0010MA in Advanced Non-Small Cell Lung Cancer	Phase 1	NCT02448251	Terminated
Hangzhou ACEA Pharmaceutical Research Co., Ltd.	Safety, Pharmacokinetic and Preliminary Efficacy Study of AC0010 in Patients with EGFR T790M Positive NSCLC	Phase 1 Phase 2	NCT02330367	Unknown status
Hangzhou ACEA Pharmaceutical Research Co., Ltd.	A Study Comparing AC0010 and Chemotherapy in Patients with Advanced NSCLC Who Have Progressed Following Prior EGFR TKI	Phase 3	NCT03058094	Withdrawn

The data were accessed on November 30, 2023 from https://clinicaltrials.gov.
